# Interactive effect of prepregnancy overweight/obesity and GDM history on prevalence of GDM in biparous women

**DOI:** 10.3389/fendo.2023.1084288

**Published:** 2023-02-16

**Authors:** Xia Xu, Feipeng Huang, Yanni Guo, Lianghui Zheng, Jianying Yan

**Affiliations:** Department of Obstetrics and Gynecology, Fujian Maternity and Child Health Hospital College of Clinical Medicine for Obstetrics & Gynecology and Pediatrics, Fujian Medical University, Fuzhou, Fujian, China

**Keywords:** gestational diabetes mellitus history, pre-pregnancy overweight/obesity, multiplicative interaction, additive interaction, recurrent gestational diabetes mellitus

## Abstract

**Background:**

Prepregnancy overweight/obesity (OWO) and gestational diabetes mellitus (GDM) history may increase the prevalence of GDM in parous women, but little is known about their potential combined effect on the prevalence of GDM in biparous women.

**Objective:**

This study aims to explore the interactive effect of prepregnancy overweight/obesity (OWO) and GDM history on the prevalence of GDM in biparous women.

**Methods:**

A retrospective study was conducted on 16,282 second-birth women who delivered a single neonate at ≧28 weeks of gestation twice. Logistic regression was used to assess the independent and multiplicative interactions of prepregnancy overweight/obesity (OWO) and GDM history on the risk of GDM in biparous women. Additive interactions were calculated using an Excel sheet that was made by Anderson to calculate relative excess risk.

**Results:**

A total of 14,998 participants were included in this study. Both prepregnancy OWO and GDM history were independently associated with an increased risk of GDM in biparous women (odds ratio (OR) = 19.225, 95% confidence interval (CI) = 17.106, 21.607 and OR = 6.826, 95% CI = 6.085, 7.656, respectively). The coexistence of prepregnancy OWO and GDM history was associated with GDM, with an adjusted OR of 1.754 (95% CI, 1.625, 1.909) compared to pregnant women without either condition. The additive interaction between prepregnancy OWO and GDM history was found to be not significant with regard to GDM in biparous women.

**Conclusions:**

Prepregnancy OWO and GDM history both increase the risk of GDM in biparous women and have multiplicative interactions but not additive interactions.

## Introduction

Gestational diabetes mellitus (GDM) is defined as carbohydrate intolerance during pregnancy that is increasing worldwide, including in China (1–3). GDM is associated with adverse pregnancy and birth outcomes, such as preeclampsia, macrosomia, birth trauma, shoulder dystocia and fetal hypoglycemia, respiratory distress, and even stillbirth (4–6). The prevalence of GDM ranged from 2.1% to 37.5% in the global population (7, 8) and 14.8% to 19.4% in the Chinese population (9, 10).

With the successful implementation of the two-child policy and three-child policy, the number of multipara women is increasing in China (11, 12). A large number of studies reported that the risk of GDM in multipara women is significantly higher than that in primipara women (13, 14). Previous studies have indicated that a variety of factors, including advanced maternal age, prepregnancy overweight or obesity, family history of GDM and T2DM, history of GDM, and subfertility or infertility was associated with GDM (15, 16). Compared with primipara, multipara women have the characteristics of old age and high BMI, which may be the possible reasons for the high incidence of GDM in multipara women. Given that most of the risk factors for GDM persist or become worse in subsequent pregnancies, it is not surprising that GDM has a high recurrence rate. A systematic review by Kim et al. (17) reported that the risk for recurrent GDM in subsequent pregnancy was as high as 30%–84% in women with prior GDM. Our previous study also reported that it was nearly 50% (18).

Maternal overweight and obesity are growing global public health concerns. The increasing prevalence of prepregnancy overweight/obesity has increased the risk of adverse maternal and neonatal outcomes, including increased rates of GDM, preeclampsia, cesarean section, and preterm delivery (19–21). The resulting increased incidence of GDM is associated with a series of adverse pregnancy outcomes.

Based on the background discussed above, both histories of GDM and overweight or obesity are independently associated with an increased risk of GDM. However, it was unclear whether women who were overweight or obese before pregnancy and had a history of GDM had a higher risk of developing GDM because the combined effect of a history of GDM and prepregnancy overweight or obesity on the prevalence of GDM in biparous women is still unknown. Therefore, in this study, we aimed to investigate whether the history of GDM and prepregnancy OWO synergistically affect the risk of GDM in biparous women.

## Methods

### The study population

The retrospective study included second-birth women who delivered a second single neonate at a gestation age of ≧28 weeks at Fujian Maternity and Child Health Hospital between January 2017 and December 2021. The eligibility criteria include all women who received perinatal care and performed a 75-g OGTT between 24 and 28 weeks of gestation. The current study was approved by the Ethics Committee of Fujian Maternity and Child Health Hospital (2020–2049). Informed consent was not required since the current study was conducted through a retrospective review of medical records.

### Data collection

All data were collected from the clinical electronic medical record and extracted into Microsoft Excel for later analysis. Data regarding demographic and obstetric characteristics were collected. The main pregnancy outcome in this study is the incidence of GDM.

### Definition

Prepregnancy body mass index (BMI) was obtained from clinic records and calculated by dividing maternal prepregnancy weight by maternal squared height. According to the adult weight standard published by the Ministry of Health of China, BMI < 18.5 kg/m^2^ is defined as underweight, 18.5 kg/m^2^ ≤ BMI < 24 kg/m^2^ is defined as normal weight, 24 kg/m^2^ ≤ BMI < 28 kg/m^2^ is defined as overweight, and BMI ≥ 28 kg/m^2^ is defined as obesity (22). All participants took a 75-g, 2-h oral glucose tolerance test. GDM was defined according to the International Association of Diabetes and Pregnancy Study Groups; a diagnosis of GDM was made when one or more of the test parameters equaled or exceeded the following cut points: fasting 5.1 mmol/L, 1-h 10.0 mmol/L, or 2-h 8.5 mmol/L (23). In this study, gestational hypertensive disorders were classified as gestational hypertension, preeclampsia, chronic hypertension with preeclampsia, and eclampsia (24). After 20 weeks of gestation, blood pressure (BP) is ≥ 140 mmHg and/or diastolic BP is ≥ 90 mmHg, but there is no proteinuria. Preeclampsia was defined as the presence of gestational hypertension with proteinuria (urine protein content > 300 mg/24 h or protein/creatinine ratio ≥ 0.3) or a systemic symptom. Eclampsia was defined as seizures that cannot be attributable to other causes in a woman with preeclampsia.

### Statistical analysis

Continuous variables were presented as median (interquartile range (IQR)), and categorical variables were presented as frequency (percentage). We compared baseline characteristics between GDM cases and non-GDM cases using the Wilcoxon two-sample test, Chi-squares test, or Fisher’s exact test.

Binary logistic regression was performed to explore the associations of prepregnancy BMI and IMH with the prevalence of GDM in multipara women. A logistic regression model was used to calculate the regression coefficients and covariance matrix for two factors at first. An Excel sheet provided by Andersson was then used to calculate relative excess risk due to interaction (RERI), attributable proportion due to interaction (AP), interaction index (synergy index (SI)), and their 95% confidence intervals (CIs) (25). The 95% CI of RERI and AP includes “0,” and the 95% CI of SI includes “1,” indicating that there is no summation interaction.

Statistical analysis was performed using IBM SPSS Statistics 26. Statistical tests were conducted on a two-sided basis, with a *p*-value of <0.05 considered statistically significant.

## Results

### Basic characteristics of the participants

**Figure 1:**
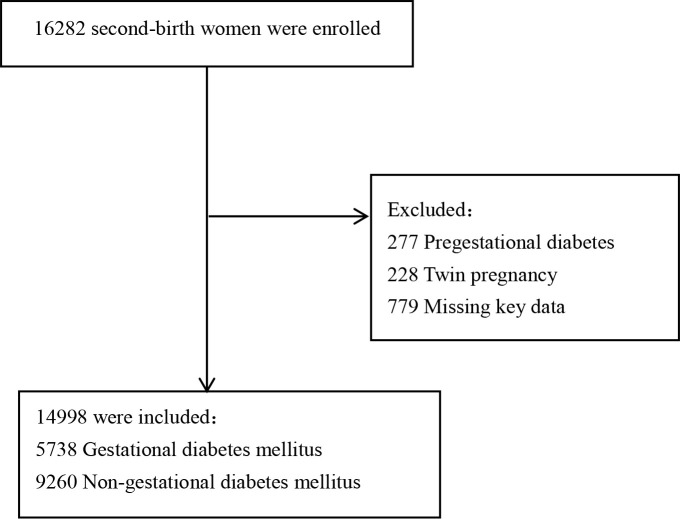
Flow chart of the study population.

A total of 16,282 women delivered their second single neonate after the gestation age of 28 weeks at Fujian Maternity and Child Health Hospital between January 2017 and December 2021. We excluded 1,284 women from the following: 277 pregnant women with prepregnancy gestational diabetes, 228 pregnant women with twin pregnancies, and 779 women who are missing data on key variables. Of the remaining 14,998 women, 5,738 were diagnosed with gestational diabetes mellitus and 9,260 were without gestational diabetes mellitus (**Figure 1**). The comparisons of baseline characteristics between gestational diabetes mellitus and nongestational diabetes mellitus groups are listed in **Table 1**. There were no significant differences in maternal age, maternal age ≧35 years, assisted reproductive techniques (ART), gestational hypertensive disorders, intrahepatic cholestasis of pregnancy, week of delivery, and mode of delivery between the two groups. Pregnant women with gestational diabetes mellitus were likely to have a higher prepregnancy BMI, birth weight, and rate of GDM history.

**Table 1:** Baseline characteristics according to the occurrence of GDM in biparous women.

Characteristics	GDM (*n* = 5,738)	Non-GDM (*n* = 9,260)	*p-*value
Maternal age (median [IQR], years)	33 [31, 36]	33 [31, 36]	0.316
Maternal age ≧35 years (No. (%))	3,594 (38.81)	2,308 (40.22)	0.086
Prepregnancy BMI (median [IQR], kg/m^2^)	21.218 [19.470, 23.310]	20.324 [18.939, 22.032]	0.000
Prepregnancy BMI (No. (%))			0.000
Underweight (No. (%))	350 (6.100)	1,764 (19.050)	
Normal weight (No. (%))	1,271 (22.150)	6,299 (68.024)	
Overweight (No. (%))	3,991 (69.554)	964 (10.410)	
Obesity (No. (%))	126 (2.196)	233 (2.516)	
GDM history (No. (%))	3,520 (61.345)	1,921 (20.745)	0.000
The interval between pregnancies (months)	29.82 ± 13.20	32.83 ± 14.64	−0.505
Gravity (No. (%))			
2	2,633 (45.887)	4,133 (44.633)	0.134
3	1,855 (32.328)	3,248 (35.076)	0.000
≧4	1,250 (21.785)	1,879 (20.292)	0.0287
ART (No. (%))	146 (2.544)	285 (3.077)	0.057
Gestational hypertensive disorders (No. (%))	519 (9.045)	324 (3.499)	0.913
Intrahepatic cholestasis of pregnancy (No. (%))	82 (1.429)	127 (1.371)	0.770
Week of delivery (median [IQR], weeks)	39.00 [38.29, 39.71]	39.00 [38.14, 39.57]	0.413
Mode of delivery (No. (%))			0.308
Vaginal birth	3,022 (52.667)	4,956 (53.521)	
Cesarean section	2,716 (47.334)	4,304 (46.490)	
Birth weight (median [IQR], weeks)	3,303 [3,019, 3,580]	3,290 [2,985, 3,560]	0.010

A Chi-square test was used to compare categorical variables, and Wilcoxon two-sample test was performed to compare continuous variables.

GDM, gestational diabetes mellitus; BMI, body mass index; OWO, overweight/obesity; ART, assisted reproductive techniques.

### Multiplicative interaction of prepregnancy BMI and history of GDM on prevalence of GDM in biparous women

Study participants were calculated based on their BMI values. The ORs of prepregnancy BMI and history of GDM for the risk of GDM in biparous women were 1.103 (95% CI, 1.086, 1.121) and 8.040 (95% CI, 4.703, 13.746) in the crude models, respectively. In the adjusted models, there were no multiplicative interactions between prepregnancy BMI and history of GDM on the prevalence of GDM in biparous women (adjusted odds ratio (aOR), 0.987; 95% CI, 0.962, 1.011). We found this combined model achieved 72.4% accuracy, 61.4% sensitivity, and 79.3% specificity (**Table 2**).

**Table 2:** The multiplicative interaction between prepregnancy BMI and GDM history for the risk of GDM in biparous women.

Category	*β*	Wold value	*p-*value	OR (95% CI)
Prepregnancy BMI	0.098	144.938	0.000	1.103 (1.086~1.121)
GDM history	2.084	58.037	0.000	8.040 (4.703~13.746)
Prepregnancy BMI by GDM history	−0.014	1.131	0.288	0.987 (0.962~1.011)

GDM, gestational diabetes mellitus; BMI, body mass index; OWO, overweight/obesity; ART, assisted reproductive techniques.

Women were classified as underweight or normal weight (BMI < 24 kg/m^2^), overweight (24 kg/m^2^ ≤ BMI < 28 kg/m^2^), and obese (BMI ≥ 28 kg/m^2^) based on their prepregnancy BMI. The ORs of prepregnancy overweight, obesity, and history of GDM for the risk of GDM in biparous women were 0.309 (95% CI, 0.226, 0.424), 7.164 (95% CI, 5.223, 9.826), and 4.758 (95% CI, 2.974, 7.613) in the crude models, respectively. In the adjusted models, there were no multiplicative interactions between prepregnancy overweight with a history of GDM and prevalence of GDM in biparous women (aOR, 1.434; 95% CI, 0.884, 2.327) as well as prepregnancy obesity with a history of GDM and prevalence of GDM in biparous women (aOR, 1.071; 95% CI, 0.651, 2.327). We found this combined model achieved 82.1% accuracy, 70.8% sensitivity, and 89.0% specificity (**Table 3**).

**Table 3:** The multiplicative interaction between stratified prepregnancy BMI and GDM history for the risk of GDM in biparous women.

Category	*β*	Wold value	*p-*value	OR (95%CI)
Prepregnancy BMI				
Underweight or normal	NA			NA
Overweight	−1.173	53.202	0.000	0.309 (0.226~0.424)
Obesity	1.969	149.133	0.000	7.164 (5.223~9.826)
GDM history	1.560	42.037	0.000	4.758 (2.974~7.613)
Overweight by GDM history	0.361	2.136	0.144	1.434 (0.884~2.327)
Obesity by GDM history	0.069	0.074	0.786	1.071 (0.651~1.762)

GDM, gestational diabetes mellitus; BMI, body mass index.

Women were then further classified as underweight or normal weight (BMI < 24 kg/m^2^) and overweight or obese (BMI ≥ 24 kg/m^2^) based on their prepregnancy BMI. The ORs of prepregnancy overweight or obesity and history of GDM for the risk of GDM in biparous women were 19.225 (95% CI, 17.106, 21.607) and 6.826 (95% CI, 6.085, 7.656) in the crude models, respectively. In the adjusted models, there were significant multiplicative interactions between prepregnancy overweight or obesity with a history of GDM and the prevalence of GDM in biparous women (aOR, 1.754; 95% CI, 1.625, 1.909). We found this combined model achieved 81.2% accuracy, 71.7% sensitivity, and 87.1% specificity (**Table 4**).

**Table 4:** The multiplicative interaction between stratified prepregnancy BMI and GDM history for the risk of GDM in biparous women.

Category	*β*	Wold value	*p*-value	OR (95%CI)
Prepregnancy BMI				
Underweight or normal	NA			NA
Overweight or obesity	2.956	2,461.092	0.000	19.225 (17.106~21.607)
GDM history	1.921	1,075.559	0.000	6.826 (6.085~7.656)
Overweight or obesity by GDM history	0.283	8.704	0.003	1.754 (1.625~1.909)

GDM, gestational diabetes mellitus; BMI, body mass index; OWO, overweight/obesity.

### Additive interaction of prepregnancy overweight or obesity and history of GDM on prevalence of GDM in biparous women

When prepregnancy overweight or obesity and a history of GDM exist at the same time, no additive interaction was found for the prevalence of GDM in biparous women (**Table 5**).

**Table 5:** Additive interaction between prepregnancy OWO and GDM history.

	RR	95% CI	SE
RERI	−1.714	−4.8970~1.469	1.624
API (%)	−36.020	−115.74~43.70	40.671
*S*	0.687	0.333~1.418	0.370

OWO, overweight/obesity.

## Discussion

In this study, we demonstrated that prepregnancy overweight or obesity and a history of GDM significantly increase the risk of GDM in biparous women separately. After accounting for confounders, the composite outcome of prepregnancy overweight or obesity and history of GDM appears to be multiplicative. However, when prepregnancy overweight or obesity and a history of GDM exist at the same time, no additive interaction was found for the risk of GDM in biparous women.

Maternal overweight or obesity before pregnancy and during pregnancy are both risk factors for GDM (26, 27). Studies suggest that it may be related to inflammation that is stimulated by a high concentration of adipokines (28). In addition, overweight or obesity may result in elevated plasma free fatty acid (FFA) levels, which leads to increased intracellular lipid accumulation in nonadipose cells like cardiomyocytes, β-cells, and hepatocytes (29). Thus, lipid accumulation in these cells induced insulin resistance *via* the activation of protein kinase C and several pathways, including diacylglycerol pathways. Clustering of these metabolic abnormalities is correlated with an increased risk of type 2 diabetes mellitus in the future (29, 30). During early pregnancy, the clustering of these metabolic risk factors has been reported to be another pathophysiology for the link between obesity and GDM.

A recent systematic review provided evidence that a history of GDM was associated with morbidity other than T2DM or cardiovascular disease and with long-term mortality (30). However, after the “One-Child Family” policy restriction was abolished and the “Two-Child Family” policy even the “Third-Child Family” policy were permitted in China, the rate of recurrent GDM may be a more important problem during follow-up after the first pregnancy for women with a history of GDM. Studies revealed that elevated circulating markers of endothelial dysfunction are present in young women with a history of GDM. Muhli et al. recently found that women with a history of GDM had a low dietary quality score and a light physical activity level during subsequent pregnancy (31). In fact, it was reported that women with a history of GDM still had a lower dietary quality and lower intensity of physical activity several years later after pregnancy (32, 33). These all explain why the history of GDM was significantly associated with a higher maternal risk for GDM recurrence.

Evidence from previous studies shows that higher prepregnancy BMI is associated with lower dietary quality, and dietary quality may decline with advancing gestation in pregnant women with obesity (34). In addition, inadequate physical activity may lead to weight gain and eventually becoming overweight and obese. Furthermore, obesity and GDM are both chronic low-grade inflammatory states (28, 35, 36). All these may be possible reasons for the multiplicative interaction, but there is no additive interaction between prepregnancy overweight or obesity and a history of GDM. For women both with a history of GDM and overweight or obesity before pregnancy, it is recommended to control their weight before pregnancy not just after pregnancy to reduce GDM risk and improve adverse pregnancy outcomes. In addition, for women with GDM, weight management should be started after the termination of pregnancy to avoid becoming overweight or obese.

In conclusion, this study explored the independent and combined effects of prepregnancy overweight/obesity and the history of GDM on the risk of GDM in biparous women, as well as their potential multiplicative and additive interactions. We found that the composite outcome of prepregnancy overweight or obesity and history of GDM appears to be multiplicative after accounting for confounders. However, when prepregnancy overweight or obesity and a history of GDM exist at the same time, no additive interaction was found for the risk of GDM in biparous women. To reduce the risk of GDM and improve perinatal outcomes, it is recommended that women with a history of GDM should try to control their weight to a normal level before pregnancy. Nevertheless, our conclusions still need to be further verified by well-designed and pregnant woman-based cohort studies.

## Data availability statement

The raw data supporting the conclusions of this article will be made available by the authors, without undue reservation.

## Ethics statement

The studies involving human participants were reviewed and approved by Ethical approval was obtained from the Fujian Maternity and Child Health Hospital Ethics Committee (2020-2049). Written informed consent for participation was not required for this study in accordance with the national legislation and the institutional requirements.

## Author contributions

All authors contributed to manuscript editing and approved the final manuscript. JY contributed to the study design. The analysis was made by XX and FH. XX and FH drafted the manuscript. YG and LZ contributed to data collection.
